# Venous Malformation in the Anterior Mediastinum

**DOI:** 10.7759/cureus.58581

**Published:** 2024-04-19

**Authors:** Hiroto Sakai, Ichiro Hasegawa, Junya Tsuzaki, Saori Okamoto, Tadayoshi Kurata

**Affiliations:** 1 Department of Diagnostic Radiology, Kawasaki Municipal Hospital, Kawasaki, JPN; 2 School of Medicine, Divison of Radiology, Keio University, Tokyo, JPN

**Keywords:** 4d-ct, hemangiomas, venous malformations, magnetic resonance imaging, diagnosis, contrast material, computed tomography, anterior mediastinum

## Abstract

Venous malformations (VMs) located in the anterior mediastinum are rare. Thus, diagnosis using imaging is often challenging, and they are typically diagnosed only after total tumor resection. Herein, we report a case of VM located in the anterior mediastinum diagnosed using computed tomography (CT) and magnetic resonance imaging (MRI). A 56-year-old woman presented for further evaluation of an anterior mediastinal mass observed during a chest CT. On CT, the mass was observed to have scattered calcifications and early and persistent enhancement with contrast material pooling dorsally in the delayed phase. On MRI, the mass was isointense on T1-weighted imaging and hyperintense on T2-weighted imaging without flow voids. From these images, we suspected the mass to be a VM, but the possibility of an arterial malformation/fistula could not be ruled out. Initially, a contrast material was injected via the arm, but to improve differentiation, it was also injected via the leg. The 4D-CT of the leg indicated no early enhancement of the mass; however, gradual enhancement was observed. This led to a definite diagnosis of VM. As she had no symptoms, we opted for a CT follow-up, and the mass remained stable for one year post-diagnosis. This case report underscores the usefulness of injecting contrast material through the leg in distinguishing VM from AVM/Fs in the anterior mediastinum.

## Introduction

Venous malformations (VMs) are the most prevalent form of vascular malformation; however, they are rarely found in the anterior mediastinum. The main treatments for VMs include sclerotherapy and resection, whereas arterial malformations/fistulas (AVMs/Fs) are primarily treated with embolization [1]. As treatment strategies depend on the type of vascular malformation, accurate diagnosis and classification are crucial. In addition, preoperative imaging is critical for determining the best surgical technique and minimizing the risk of complications. A case of VM of the anterior mediastinum that was suspected to be a thymoma on preoperative imaging and resulted in massive intraoperative bleeding (1500 ml) has been reported [2]. Herein, we present an unusual case involving a symptom-free adult with a VM located in the anterior mediastinum, diagnosed using dynamic enhanced computed tomography (CT) with contrast material administered through both arms and legs.

## Case presentation

**Figure 1:**
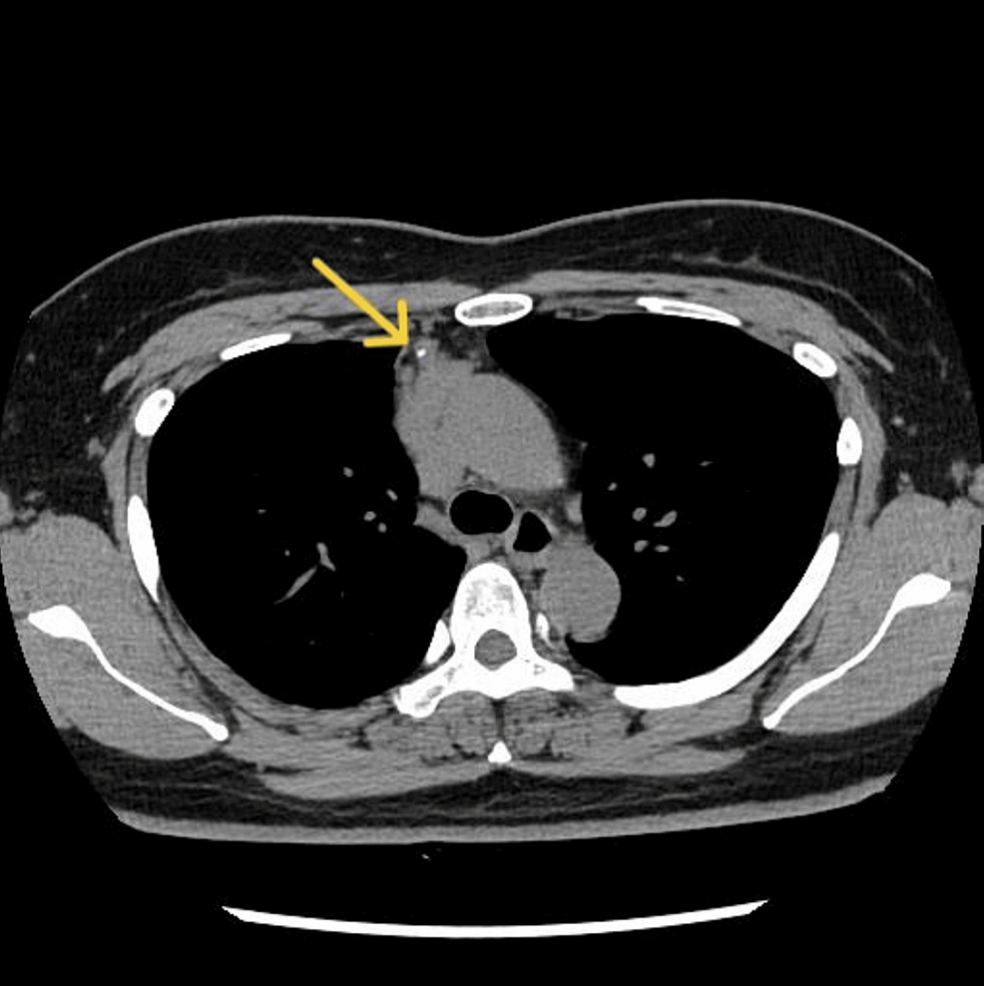
Unenhanced compyted tomography (CT) showing a lobulated mass with sparse calcification in the anterior mediastinum.

**Figure 2:**
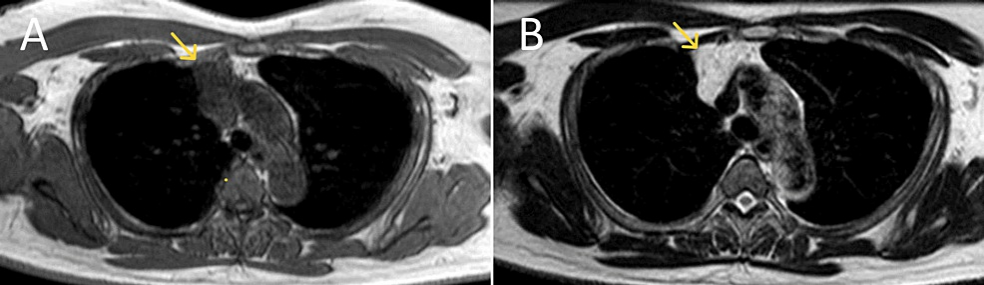
T1-weighted imaging (T1WI) and T2-weighted imaging (T2WI) MRI The mass appeared isointense on T1WI (A) and hyperintense on T2WI (B), with no observed flow voids.

**Figure 3:**
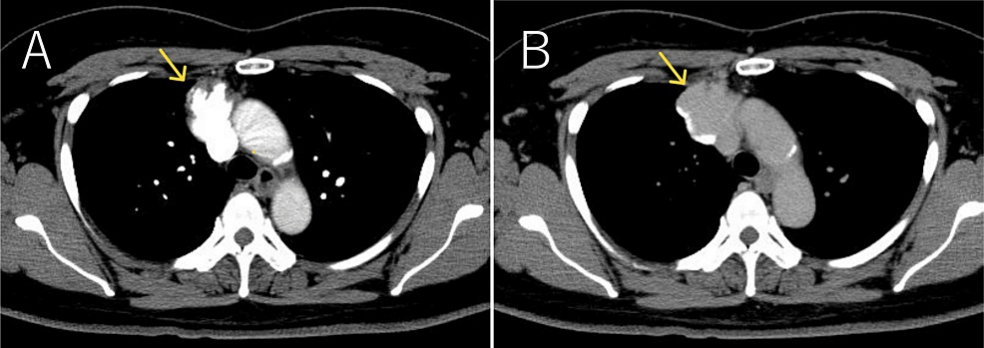
Dynamic contrast-enhanced CT (injected via the arm) Dynamic contrast-enhanced CT injected via the arm showed early and persistent enhancement of the mass, with contrast material pooling on the dorsal side in the delayed phase.

A 56-year-old woman visited our hospital for evaluation due to an anterior mediastinal mass discovered on chest CT, which was a new finding. Despite having no symptoms, an unenhanced CT showed an anterior mediastinal mass with sparse calcification (Figure 1). On MRI, the mass appeared isointense on T1-weighted imaging (T1WI) and hyperintense on T2-weighted imaging (T2WI) without flow voids (Figure 2). Dynamic enhanced CT later revealed early and persistent enhancement with contrast material accumulation in the delayed phase, but the arterial inflow of the contrast remained unknown (Figure 3). While the imaging suggested a VM, the possibility of an AVM/F could not be ruled out due to the early enhancement observed.

**Figure 4:**
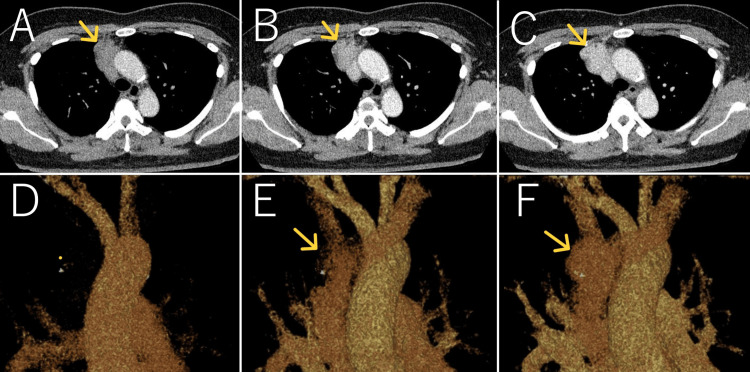
Dynamic contrast-enhanced computed tomography (CT) and 4D-CT (injected via the leg) Dynamic contrast-enhanced computed tomography (CT) and 4D-CT injected via the leg. (A) and (D) are 1 second, (B) and (E) are 12 seconds, (C) and (F) are 16 seconds after starting the scan. The mass exhibited no enhancement in the early phase but showed a gradual increase and persistent enhancement after the contrast material reached the left brachiocephalic vein.

Initially, a contrast material was injected via the arm, but to improve differentiation, injection via the leg was also performed. We conducted four-dimensional CT (4D-CT) using a 256-row detector CT system with the test bolus tracking method. We injected the test bolus for 2 seconds, followed by a 0.9% saline solution for another 4 seconds. After waiting for 20 seconds, the main bolus injection for 16 seconds was started, followed by the injection of a saline solution for 4 seconds. This image acquisition consisted of 37 intermittent volume scans with a scan interval of 0.5 seconds. We inserted a 20-gauge catheter into the right cutaneous vein of the lower leg with an injection flow rate of 5 mL/s. The CT dose-length product was 1157.01 mGy·cm. The 4D-CT of the leg showed no early enhancement of the mass, instead displaying gradual enhancement (Figure 4), which led to a definite diagnosis of VM. Subsequent CT follow-up a year later confirmed the stability of the mass post-diagnosis. 

## Discussion

The International Society for the Study of Vascular Anomalies (ISSVA) primarily classifies vascular anomalies as vascular tumors or malformations [3]. Vascular tumors involve endothelial cell proliferation, whereas vascular malformations do not. This classification is becoming increasingly standardized internationally. VMs are categorized as simple vascular malformations and placed in the slow-flow malformation category by the ISSVA. However, historical challenges in identifying and classifying vascular anomalies have arisen because of the confusing nomenclature [4]. VMs and hemangiomas (venous, cavernous, and capillary) are often described interchangeably in the medical literature. Venous hemangiomas exhibit thickened vascular smooth muscle and a dilated vascular lumen, whereas cavernous hemangiomas lack vascular smooth muscles and capillary hemangiomas have a small vascular lumen. The size of the intratumoral vascular lumen varies depending on the hemangioma subtype [5]. In the mediastinum, VMs and hemangiomas are sometimes used synonymously, with most reports referring to them as hemangiomas. VMs are commonly found in the head and neck region (40%), extremities (40%), and trunk (20%) [6], accounting for <0.5% of all mediastinal tumors [7]. Cavernous hemangiomas constitute the largest proportion of all hemangiomas.

Imaging diagnosis poses a challenge because of its rarity and nonspecific clinical manifestations, often leading to diagnosis after total tumor resection [8]. VMs undergo a continuous cycle of spontaneous thrombosis and thrombolysis, with persistent thrombi potentially calcifying and forming phleboliths, which are considered pathognomonic but present in only 30% of radiographs [6,9]. CT is more sensitive in detecting calcifications. On enhanced CT, VMs in other body regions display gradual and persistent mass enhancement owing to slow blood flow [10]. Various enhancement patterns may occur in the anterior mediastinum, including central, mixed central and peripheral, peripheral, and nonspecific increased attenuation [11]. A previous report described a capillary hemangioma connected to the left brachiocephalic vein exhibiting central and peripheral patterns on CT [12]. In this case, we suspected the mass to be a VM due to the presence of phleboliths and marked central and peripheral enhancement continuing from the early to delayed phases, with contrast material pooling on the dorsal side due to delayed washout. However, we could not determine whether the early enhancement resulted from a feeder from an artery or vein. Injecting contrast material via the arm could lead to strong enhancement in the early phase if it is connected to the brachiocephalic or superior vena cava that flows backward, indicating the possibility of AVM/F.

To aid in differentiation, we propose injecting the contrast material via the leg. To the best of our knowledge, the dynamic features of VMs in the anterior mediastinum following leg injections have not yet been reported. We conducted 4D-CT using a 256-row detector CT system with the test bolus tracking method by injecting the test bolus via the leg. The mass exhibited no enhancement in the arterial phase but showed gradual and persistent enhancement after the contrast reached the brachiocephalic vein or superior vena cava. These features resemble those of VMs found in other body regions. Combining these findings with the previous CT results, we diagnosed the mass as a VM. As the patient had no symptoms, we opted for CT follow-up, and the mass remained stable for one year post-diagnosis.

## Conclusions

We report on an adult with an asymptomatic VM in the anterior mediastinum. Owing to its rarity, diagnosing it through imaging is often challenging. Our case indicated that injecting a contrast material through the leg might be useful in distinguishing VM from AVM/Fs in the anterior mediastinum. Based on the information provided, further studies can explore the use of imaging techniques, such as injecting contrast material via the leg, to differentiate between VM and AVM/Fs in the anterior mediastinum. This could potentially contribute valuable insights to the field and improve diagnostic accuracy for similar cases in the future.
